# Craniofacial manifestations in osteogenesis imperfecta type III in South Africa

**DOI:** 10.1038/bdjopen.2017.21

**Published:** 2017-10-20

**Authors:** Manogari Chetty, Tina Sharon Roberts, Lawrence Stephen, Peter Beighton

**Affiliations:** 1 Faculty of Dentistry, University of the Western Cape, Cape Town, South Africa; 2 Division of Human Genetics, Faculty of Health Sciences, University of Cape Town, Cape Town, South Africa; 3 University of the Western Cape/University of Cape Town Collaborative Dental Genetics Clinic, Red Cross Childrens Hospital, Cape Town, South Africa

## Abstract

**Objectives:**

Osteogenesis imperfecta type III (OMIM 259420) is a severe autosomal recessive disorder. Affected individuals have multiple fractures, develop limb deformities with spinal malalignment and stunted stature.

**Materials and methods:**

The frequency of Osteogenesis imperfecta type III (OI III) is relatively high in the indigenous Black African population of South Africa. A review of the literature revealed a paucity of information regarding the craniofacial manifestations of the disorder in this ethnic group. The findings in 64 affected persons are documented.

**Results:**

These abnormalities are related to the abnormal bone matrix which results in a deformed skull and dental malocclusion. The physiological process of swallowing may be an aetiological factor in the progressive development of a flattened palate. Mild changes in the shape of the head of the mandibular condyle and a lack of cortical bone on the joint surfaces were observed on cone beam computed tomography (CBCT) images. Affected persons had marked variations in the paranasal sinuses, including sinus hypoplasia and partial opacification. Cranial base anomalies were diagnosed from cephalometric radiographs and lateral skull radiographs. Platybasia and a ‘J’ shaped sella turcica were observed.

**Conclusion:**

The craniofacial abnormalities emphasize the importance of a raised level of awareness in terms of dental management and the challenges.

## Introduction

Osteogenesis imperfecta type III (OI III) (OMIM 259420) is a severe autosomal recessive (AR) disorder in which frequent fractures and progressive limb and spinal deformity result in profound physical disability. The condition is clinically and genetically heterogeneous and maxillofacial and dental manifestations have significant implications in terms of management in some forms of the disorder.

In 1979, in Australia, Sillence *et al.*^1^ found OI III to be about one-eighth as frequent as AD OI with blue sclerae. Among 345 pedigrees with OI, Sillence *et al.*^2^ found 7 that had autosomal recessive inheritance with criteria suggestive of type III OI such as clinically normal teeth and sclerae with fractures or deformability present from birth.

At the most recent meeting of the Nosology Committee of International Skeletal Dysplasia Society, the 9th edition of the nosology lists 436 disorders in 42 groups and 364 genes.^3^ OI remains in group 25 and the phenotypically based Sillence classification is maintained as OI III being of AD as well as AR mode of inheritance and 15 determinant genes are listed.^3,4^

Although worldwide, autosomal recessive OI is rare, it has emerged that the frequency of a form of OI III is relatively high in the indigenous Black African population of South Africa (SA).^5–7^ There is a paucity of information in the literature regarding the dental and craniofacial features of the disorder in this ethnic group. For these reasons, an investigation of these manifestations in 64 individuals with OI III in this population was undertaken.

The aim and objectives of this study was to document and elucidate the dental and craniofacial manifestations in persons with OI III in order to identify the dental needs of the affected persons and to facilitate the formulation of appropriate protocols for dental management in SA.

The craniofacial findings have implications for dental management and they are presented, depicted and discussed in this article.

## Patients and methods

### Ethics

All investigations were undertaken in complete accordance with the Declaration of Helsinki, the Hippocratic Oath and the Singapore Statement on Research Integrity. Formal ethical approval (HREC reference number: 203/2013) was obtained from the University of Cape Town’s ethics committee.

With the co-operation of medical colleagues in different centres, a series of 64 Black African individuals with a confirmed diagnosis of OI III in South Africa, with ages ranging from 3 months to 30 years, were assessed as a component of a PhD investigation by MC at the University of Cape Town. This study had a predominant clinical component which involved a craniofacial and dental examination of the affected persons and which were subsequently documented. When necessary, imaging studies were undertaken. Craniofacial imaging was undertaken when this resource was locally available. Panorex and cephalometric radiographs were obtained and cone beam CT imaging was also carried out when appropriate. The attainment of radiographic images were dependent on the necessity of these investigations in terms of clinical management and availability of these facilities. Radiographic images that had previously been obtained by the patient’s physician, for instance, lateral views of the skull, were also available to the authors. The imaging facilities used were only available at specific dental centres. Although radiographic resources were limited, 15 CBCT images, 20 panorex and 20 cephalometric radiographs were obtained. All radiographic findings were confirmed by two consultant radiologists from the universities of the Western Cape and Stellenbosch.

Biological material from these affected persons was analyzed for a mutation in the *FKBP10* gene and 27 persons had a positive result. The molecular findings are not commented on further as they are the subject of a focused genetic and molecular publication.^8^

In the illustrations affected individuals are represented by alphabetical-numerical designations pertaining to the investigation center and the chronological order in which they were assessed. In the text, for the sake of clarity the authors have commented on the craniofacial manifestations following the documentation of the findings of each specific anatomical region.

## Results and comments

### Occlusion

In this project, occlusion was determined from a clinical oro-dental examination of the affected persons. When possible, cephalometric radiographs were obtained in order to confirm a skeletal-jaw relationship. Cephalometrics is the interpretation of lateral skull radiographs taken under standardized conditions.

Abnormalities in the occlusion and skeletal relationship of the upper and lower jaws are presented and summarized in Table 1. The occlusion was assessed and classified from a craniofacial and dental clinical examination and when available, an interpretation of a cephalometric radiograph. These malocclusions were classified according to the Angle classification.

Other clinical factors that influence the occlusion of an individual such as open, cross, edge to edge bites and splayed (proclined) teeth were also recorded in Table 1.

An edge to edge bite in this instance was only identified in children with only their deciduous dentition and in those in the mixed dentition period.

**Figure 1:**
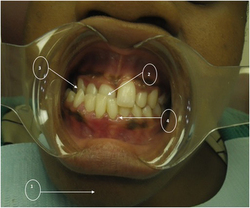
Intraoral photograph of CPT 1. She has a skeletal Class III malocclusion with a prognathic mandible (1) and an anterior crossbite (2). A posterior crossbite (3) and swollen, inflamed gingiva (4) are evident.

A clinical image of CPT 1 (Figure 1) is presented in order to depict the occlusal abnormalities observed.

**Figure 2:**
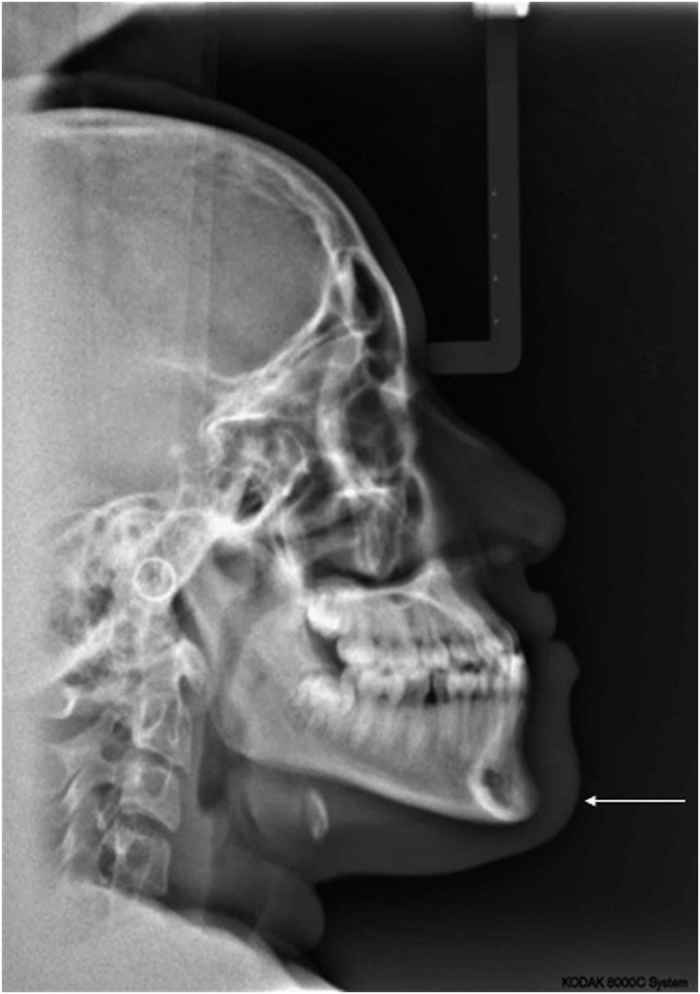
Cephalometric radiograph of affected person CPT 1 confirming a skeletal Class III malocclusion. Her mandible is prognathic (arrow).

Several anomalies in terms of malocclusion are illustrated in Figure 1 of CPT 1. A cephalometric radiograph (Figure 2), obtained prior to the commencement of orthodontic treatment, confirms the presence of a dental Class III and skeletal Class III malocclusion in CPT 1.

An edge to edge bite can be observed in children who have only their primary dentition. This occlusal observation is significant as it often tends to progress to an adult Class III dental malocclusion and a potential skeletal Class III malocclusion.

### Palatal anatomy

The anatomy of the palate is clinically relevant since it is a contributing factor to the development of cross bites and open bites and in this manner, impacts on the occlusal status of an affected person.

During the course of the clinical investigations, the author observed flattened palates in several young adult persons with OI III. Conversely, the palatal anatomy of many young children was within normal limits. In view of this disparity, the palatal anatomy of affected individuals was documented using subjective scores where 1 indicated a high arched palate, 2 suggested a palate within normal limits and 3 represented a flattened palate.

Thirty-nine individuals had flat palates and 25 persons had palates that were within normal limits.

The persons with the flattened palates were predominantly early adolescents and teenagers. The individuals with normal palates were children aged 8 years and below.

**Figure 3:**
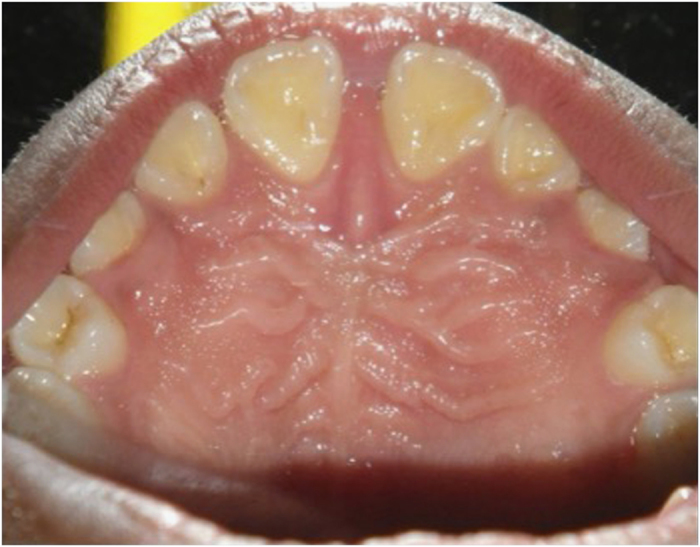
Flat palate of PMB 1 at 18 years. Her anterior teeth are splayed (proclined).

**Figure 4:**
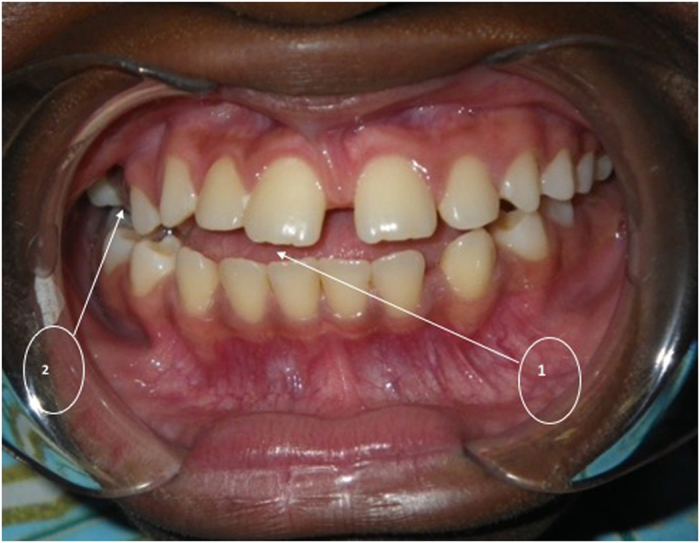
An intraoral picture of PMB 1. She has an anterior open bite (1) and a posterior cross bite (2).

Clinical images of PMB 1 are presented below in order to depict a flat palate with consequent occlusal abnormalities (Figures 3 and 4).

#### Comment: Occlusion and palatal anatomy

Significant dental malocclusion, particularly Class III, was noted in OI III affected Black African persons.

The craniofacial abnormalities are related to the abnormal bone matrix and consequent skeletal malleability which leads to a deformed skull. The affected persons’ posture, weight and size of the head are often abnormal and these variables may contribute to the development of the Skeletal Class III malocclusion. A mandibular overjet (prognathic mandible) in individuals with OI III has been previously documented.^9^ Cephalometric radiographs have revealed a flattened cranial base, a posteriorly reclined maxilla and a protrusive mandible, thereby creating a profile of midface hypoplasia. This process tends to occur in adolescence and early adulthood.^10,11^

Some forms of malocclusion are secondary and develop due to dental attrition and loss of vertical dimension of maxillofacial structures. Attrition may lead to the development of a deep bite or an anterior rotation of the mandible and a subsequent mandibular overjet.^12^ An edge to edge bite in children often develops into an adult Class III malocclusion.^13^

The increased incidence of anterior and posterior cross-bites and anterior and posterior open-bites is to be expected given the high incidence of Class III malocclusion. Posterior open-bites occurred in adolescence and young adults and were often bilateral. These posterior open bites can be explained by an abnormal vertical dento-alveolar development thus permitting an increased interdental space for the tongue. Posterior open-bites can also be caused by the absence of dental compensation for the protrusive mandible. Anterior open-bites do not allow the affected person to incise food adequately and posterior cross bites and open bites compromises an individual’s ability to masticate.

During the oral phase of swallowing, food is moved against the hard palate and the tip of the tongue is placed on the palate behind the incisor teeth. Food is then moved posteriorly into the pharynx by the elevation and pressure of the anterior two thirds of the tongue against the palate. As the child gets older, this physiological process of swallowing and consequent tongue pressure against the palate may be an aetiological factor in the progressive development of a flattened palate. In turn, the flattening of the palate could also be a contributory factor to the development of cross bites and open bites.

The pressure of the tongue may also lead to changes in the axial inclination of the incisors and subsequent splaying of the teeth. Since the affected individuals had a decrease in the quantity and quality of their bone, forces such as tongue positional pressures can result in a deformity of the palate and alveolar bone.

In order to identify the many factors that may contribute to the malocclusion, longitudinal studies would be necessary in OI III affected persons in SA.

### Periodontal status

Bitewing intraoral radiographs are ideal in the radiological assessment of the periodontium, but, it was imperative to minimize the radiation exposure levels in every instance. For this reason, available panorex and CBCT images were examined and periodontal findings were reported.

Affected individuals did not present with an unequivocal increase in susceptibility to periodontal disease even though a loss of their lamina dura was evident on dental radiographs. These findings are similar to those reported in the literature.^9,14^

Thirteen affected individuals between 16 and 30 years exhibited an elevated plaque and gingival index with an average pocket depth of 5 mm. An estimate of 2 mm of alveolar bone loss was detected in five individuals in whom radiographs were available.

Further longitudinal studies are necessary in OI III affected individuals in order to determine the incidence and progression of periodontal disease in SA.

### Temporo-mandibular joint

**Figure 5:**
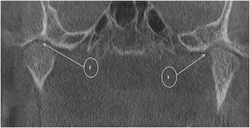
Coronal section through Cone Beam Computer Tomography of DBN 4. The heads of the mandibular condyles are irregularly shaped (1) and there is mild surface irregularity with loss of surface cortical bone (2).

No significant TMJ findings were observed in the majority of the project participants. Nevertheless, mild changes in the shape of the head of the mandibular condyle and a lack of cortical bone on the joint surfaces were observed on CBCT images of 5 individuals between the ages of 15 and 20 years (Figure 5). It is likely that TMJ problems may arise later in adulthood in these persons.

Thirteen individuals between 15 and 20 years were clinically examined and all of them revealed a maximum oral cavity opening of <35 mm. It is relevant that the normal range of opening is 50–60 mm depending on the age and size of the individual.

Their maximum lateral movement of the mandible was between 5–6 mm. The normal average range of lateral movement to the right or left is between 7 and 12 mm. The maximum protrusive movement of the affected persons was 5 mm. The normal range of protrusive movement is between 8 and 11 mm depending on the size of the individual and the skull morphology.

#### Comment: TMJ

Their limited oral opening was most likely due to the irregularly shaped mandibular condyles.

To the best of the author's knowledge, there are no reports on the involvement of the TMJ in OI III in SA. Longitudinal studies would be necessary in OI III affected individuals in order to determine the possibility of further TMJ disorders developing.

### Sinuses

**Figure 6:**
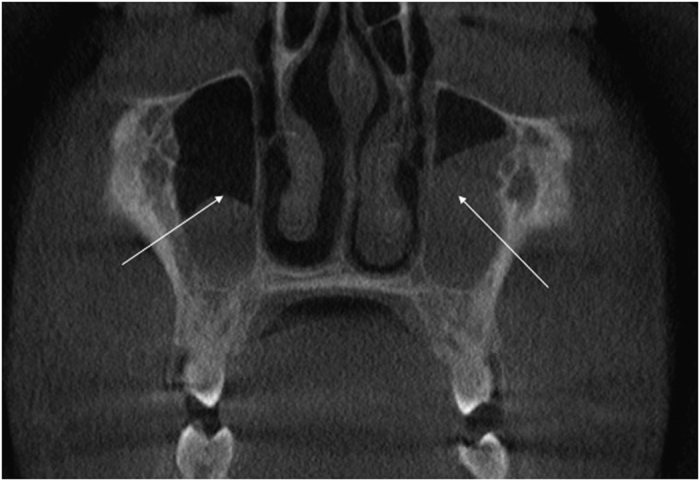
Partial opacification of the L and R maxillary sinuses (arrows) of DBN 4.

In the five affected individuals in whom CBCT images were obtained, marked changes were evident in several paranasal sinuses, notably sinus hypoplasia and partial opacification (Figure 6). These five individuals also gave a history of repeated upper respiratory tract infections and they experienced mild to moderate difficulty in breathing through their nose.

#### Comment: Sinuses

To the best of the author’s knowledge, there are no published reports on sinus changes or complications in persons with OI III and the significance of these sinus observations are uncertain.

### Cranial base abnormalities

Several reports have documented cranial base anomalies in OI individuals.^15–17^ Since pathology in the craniocervical junction can be a cause of serious complications in OI affected individuals, irregularities in this area were noted when recognized in this project. Cranial base anomalies can be diagnosed from cephalometric radiographs and lateral skull radiographs. In each instance, a consultant radiologist identified and verified these findings.

**Figure 7:**
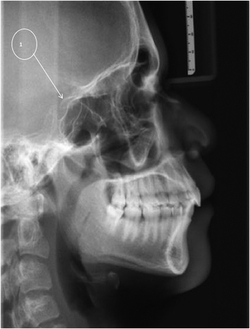
A cephalometric radiograph of DBN 4. Platybasia and a ‘J’ shaped sella turcica are evident (1).

In order to illustrate these observations a cephalometric radiograph of DBN 4 (Figure 7) was assessed. Features of platybasia and a ‘J’ shaped sella turcica were observed.

A further cephalometric radiograph of DBN 2 was also assessed. He also had platybasia and a ‘J’ shaped sella turcica.

Cephalometric radiographs of five Black African affected persons were obtained as requested by the orthodontist to whom they were referred and a further six individuals already had lateral skull radiographs available. The radiographs of these 11 Black African affected persons aged between 15 and 21 years were analyzed.

Features of platybasia and a ‘J’ shaped sella turcica were noted in 8 individuals.

#### Comment: Cranial base abnormalities

In OI III, reports suggest that pathology of the craniocervical and base of skull region can be divided into platybasia, basilar invagination and basilar impression and it is suggested that these complications can occur separately or concurrently.^15–17^ Cranial base anomalies impact on dental therapy in that caution is warranted when a patient’s head is manipulated in order to avoid atlanto-axial subluxation and spinal cord compression.

Arponen *et al.*^15^ documented the natural progression of cranial base anomalies in 150 persons with OI aged between 0 and 39 years and reported that 37% had abnormalities. These authors found that the severity of the condition and marked growth failure suggested the presence of cranial base anomalies. They subsequently recommended a radiological management strategy with regular follow-up. In their cohort of patients, the number of individuals on bisphosphonate therapy was low; hence, a further study was undertaken in order to resolve the issue of the effect of bisphosphonates in the development of cranial base anomalies.^16^ They reported that although the early initiation of bisphosphonate treatment may defer the development of cranial base pathology, abnormalities also arise despite bisphosphonate therapy.

In severe forms of OI, Sillence^18^ advocated radiographic screening of the skull base every 2 or 3 years from the age of 5 years onwards.

In addition to platybasia, a’J’ shaped sella tursica was observed in 8 individuals in this survey in whom radiographic access was available. In cephalometric analyses of dentofacial morphology, the sella point constitutes an important reference point. This feature warrants awareness by the dental clinician in order to enable the distinction between pathology and normal developmental patterns.

An altered shape of the sella can be present in normal persons as well as in medically compromised individuals with spina bifida^19^ and craniofacial deformities.^20,21^

The shape of the sella has been observed by researchers in 180 persons between the ages of 11 years and 26 years. A significant difference was observed in the shape and diameter of the sella in persons with a skeletal Class I, Class II and Class III malocclusion.^22,23^ Since the morphology of the sella turcica may vary from individual to individual, and the establishment of normal standards will aid in the process of eliminating any abnormality in such an important region. The sella shape and dimensions reported in this study^22^ may be used as a reference standard for further investigations involving the sella turcica area in Saudi subjects.

To the best of the author’s knowledge, there is no reported association between a ‘J’ shaped sella turcica and OI but the author considers this a noteworthy finding despite an asymptomatic presentation. It may be relevant that a ‘J’ shaped sella tursica has been reported in Hajdu-Cheney syndrome, another rare genetic disorder of the skeleton.^24–26^ This condition resembles OI by virtue of osteoporosis and craniofacial features of platybasia, micrognathia, premature loss of teeth, wormian bones and open cranial sutures.

## Conclusion

The craniofacial abnormalities in OI III affected persons impact on their dentofacial appearance and masticatory function. A detailed dental and craniofacial investigation is necessary in affected persons in order to identify any primary or secondary abnormalities. Short stature, shortness of the neck, limited opening of the mouth and mobility make procedures, such as radiographic examination, impression taking, bonding of orthodontic brackets and dental material and oral hygiene, exigent for both the patient and the dental practitioner.

Appropriate treatment planning for the management of individuals with OI III should, ideally, involve communication between dental specialists in the fields of paediatric dentistry, periodontics, orthodontics, oral surgery, oral radiology and prosthodontics. Unfortunately, economic constraints and the lack of appropriate dental facilities precluded necessary and adequate management and resources at every level are limited to virtually non existant in many areas.

In SA, the development of facilities for the management of these affected persons, particularly adolescent and adult individuals, warrants attention especially in terms of the provision of resources at a socio-political level. In the face of a burgeoning population, it is hoped that this deficiency is recognized and addressed in the context of national health care.

## Figures and Tables

**Table 1:** Summary of Occlusal Findings

*Class I*	*Class II*	*Class III*	*Anterior open bite*	*Posterior open bite*	*Anterior cross bite*	*Posterior cross bite*	*Edge to edge bite*	*Proclined teeth*
10	0	26	11	5	7	18	19	14
